# Temporally adaptive acoustic sampling to maximize detection across a suite of focal wildlife species

**DOI:** 10.1002/ece3.5579

**Published:** 2019-08-22

**Authors:** Cathleen Balantic, Therese Donovan

**Affiliations:** ^1^ Vermont Cooperative Fish and Wildlife Research Unit Rubenstein School of Environment and Natural Resources University of Vermont Burlington VT USA; ^2^ U.S. Geological Survey Vermont Cooperative Fish and Wildlife Research Unit Rubenstein School of Environment and Natural Resources University of Vermont Burlington VT USA

**Keywords:** adaptive sampling, automated acoustic monitoring, bioacoustics, detection probability, occupancy, optimization, wildlife

## Abstract

1. Acoustic recordings of the environment can produce species presence–absence data for characterizing populations of sound‐producing wildlife over multiple spatial scales. If a species is present at a site but does not vocalize during a scheduled audio recording survey, researchers may incorrectly conclude that the species is absent (“false negative”). The risk of false negatives is compounded when audio devices have sampling constraints, do not record continuously, and must be manually scheduled to operate at pre‐selected times of day, particularly when research programs target multiple species with acoustic availability that varies across temporal conditions.
2. We developed a temporally adaptive acoustic sampling algorithm to maximize detection probabilities for a suite of focal species amid sampling constraints. The algorithm combines user‐supplied species vocalization models with site‐specific weather forecasts to set an optimized sampling schedule for the following day. To test our algorithm, we simulated hourly vocalization probabilities for a suite of focal species in a hypothetical monitoring area for the year 2016. We conducted a factorial experiment that sampled from the 2016 acoustic environment to compare the probability of acoustic detection by a fixed (stationary) schedule versus a temporally adaptive optimized schedule under several sampling efforts and monitoring durations.
3. We found that over the course of a study season, the probability of acoustically capturing a focal species (given presence) at least once via automated acoustic monitoring was greater (and acoustic capture occurred earlier in the season) when using the temporally adaptive optimized schedule as compared to a fixed schedule.
4. The advantages of a temporally adaptive optimized acoustic sampling schedule are magnified when a study duration is short, sampling effort is low, and/or species acoustic availability is minimal. This methodology presents the opportunity to maximize acoustic monitoring sampling efforts amid constraints.

## Graphical abstract

Acoustic recordings of the environment can produce data for characterizing populations of sound‐producing wildlife over multiple spatial scales. We developed a temporally adaptive acoustic sampling algorithm to maximize detection probabilities for a suite of focal species amid sampling constraints. Using a simulation study, we found that over the course of a study season, the probability of acoustically capturing a focal species at least once via automated acoustic monitoring was greater (and acoustic capture occurred earlier in the season) when using the temporally adaptive optimized schedule as compared to a fixed schedule.

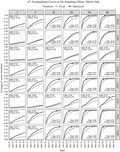

## 1 INTRODUCTION

Automated remote acoustic monitoring of wildlife offers a means to characterize the distribution of sound‐producing species—such as birds, amphibians, bats, and insects—across vast landscapes (Dawson & Efford, 2009; Marques et al., 2013). Because acquiring species abundance data is often logistically impractical at large spatial scales, research programs may instead collect species detection–nondetection data, an endeavor with which automated remote acoustic monitoring is compatible (Cerqueira & Aide, 2016; Furnas & Callas, 2014). In a typical passive remote acoustic monitoring program, audio recording devices deployed at fixed locations take environmental recordings based on a schedule that has been manually input to the device. Commercially available recording units often store recordings directly on the device (e.g., Wildlife Acoustics, 2016), which obligates the researcher to be physically present to retrieve data from a storage card. Alternatively, recordings units may expedite data access and analysis by sending files in near‐real time to a server using a cellular or Wi‐Fi network (McKown, Lukac, Borker, Tershy, & Croll, 2012; ARBIMON: Aide et al., 2013; Balantic & Donovan, 2019a; Gage & Farina, 2017). A drawback of using the cellular network to transmit audio files is that data plans can be costly and may constrain the amount of acoustic sampling that is possible, which provided the primary motivation for this work.

Regardless of whether audio recordings are stored on board the device or transmitted automatically over a network, remote acoustic monitoring of wildlife presents a multitude of opportunities and challenges (Gibb, Browning, Glover‐Kapfer, & Jones, 2019). Monitoring programs can collect massive volumes of audio data—often too much for researchers to listen to and examine manually. Methodologies that permit automated detection of target sounds from audio recordings offer a means for coping with large data volumes, but can be fraught with detection mistakes (Shonfield & Bayne, 2017). Automated detection methods may fail to detect sounds issued by species of interest (false negatives), or mistakenly detect false alarms not issued by the target species (false positives; Acevedo, Corrada‐Bravo, Corrada‐Bravo, Villanueva‐Rivera, & Aide, 2009; Balantic & Donovan, 2019a; Buxton & Jones, 2012; Duan et al., 2013; Marques et al., 2013). Occupancy modeling frameworks are a well‐established approach for accommodating the detection mistakes that arise from remote acoustic monitoring and can deal both with false negatives (Cerqueira & Aide, 2016; Furnas & Callas, 2014; Rich, Beissinger, Brashares, & Furnas, 2019) and false positives (Balantic & Donovan, 2019b; Banner et al., 2018; Chambert, Miller, & Nichols, 2015; Chambert, Waddle, Miller, Walls, & Nichols, 2018). However, false negatives due to suboptimal automated detection methodologies are distinct from false negatives that occur as a consequence of deficient audio sampling schedules. Research programs with limited audio sampling capacity may benefit from methods that maximize target detection probabilities given that a target species is present.

The methodology we outline in this work arose from our real field experience implementing a remote acoustic monitoring study. We deployed a proof‐of‐concept remote acoustic monitoring program in the Colorado‐Sonoran Desert of California, on Bureau of Land Management (BLM) public land (Balantic & Donovan, 2019a). We installed stationary smartphone‐based acoustic monitoring units at 16 sites within the BLM‐managed Riverside East Solar Energy Zone, a 599‐km^2^ parcel designated for utility‐scale solar energy development. As a pilot study, the work focused on development of smartphone‐based monitoring methodology rather than ecological inference; monitoring locations were selected in microphyll woodlands to record songbirds, and in historical breeding pond locations in hopes of recording Couch's Spadefoot (*Scaphiopus couchii*), an amphibian whose current distribution status across the area is unknown. The use of smartphones for near real‐time data transmission, in combination with our limited research budget, constrained us to taking *n* = 9 total minutes of recordings per monitoring location per day. The constraints imposed by data transmission costs were outweighed by the logistical benefit of having access to our audio data the day after it was recorded, despite our physical location over 4,000 km from the study site.

The difficulty with manually setting a recording schedule to survey wildlife is that truly present species do not always provide an acoustic cue during the recording session. If a species is present but does not announce itself during scheduled recording periods, the species is logged as absent, resulting in a false negative (MacKenzie et al., 2002). Across time and space, deficient fixed recording schedules can fail to adequately describe a pattern of occupancy, potentially resulting in conservation management decisions that are at odds with management objectives. For example, if an amphibian species of interest only vocalizes after the first substantial rainfall of the season, as is the case for Couch's Spadefoot (*S. couchii*) in the Sonoran Desert (Mayhew, 1965), and no recordings were scheduled at a time that captures this event, then researchers may conclude the species is likely absent. Resource managers may subsequently use this information to make land use decisions that unwittingly sabotage their own conservation goals. As such, low species detection probabilities motivate the development of sampling protocols that improve the chances of detecting a species given that it is present (MacKenzie et al., 2006).

The task of avoiding false negatives is magnified when large‐scale acoustic monitoring regimes attempt to track multiple focal species available under varying conditions (Manley, Zielinski, Schlesinger, & Mori, 2004; McKown, 2012). Focal species may have diverse behaviors and life histories, driving vocalization activity patterns that vary across time of day, time of year, and weather conditions. For example, a comprehensive monitoring program may be interested in tracking the occurrence patterns of breeding birds that vocalize on spring mornings with minimal rain and wind, seasonally available amphibians that only vocalize after fall monsoon rains, and nocturnally active species such as nightjars (Caprimulgidae family) or coyotes (Canidae family), as was the case in our pilot monitoring program. Certain species within the focal set may be of special concern and therefore merit higher monitoring priority. Thus, remote acoustic monitoring programs targeting multiple species face the prospect of low detection probabilities for some or all targets if using a fixed, manually applied schedule for sampling, particularly if sampling is constrained by mobile data transmission costs.

Alongside detection challenges, acoustic monitoring programs often encounter constraints that restrict sampling efforts, prompting the need for guidance in the development of effective sampling schedules that avoid squandering key resources. Contingent on program circumstances, budget and logistical limitations may curb the total number of allowable audio samples, total amount of sampling time, and sample file sizes for storage or efficient transfer over a mobile or Wi‐Fi network (Gage, Joo, Kasten, Fox, & Biswas, 2015). Even if a Wi‐Fi or cellular network is available to facilitate the real‐time transmission of audio recordings (allowing researchers to avoid collecting recordings from on‐site memory cards), some portion of the research budget is required to support the Wi‐Fi or cellular data plan, which may limit the total recording time that can be taken and transmitted over the network. Additionally, if network signal is weak, it is prudent to limit recordings to short intervals of time (~1–2 min) to ensure efficient and reliable file transmission over the network, particularly if using high sampling rates (44.1 + kHz) and/or uncompressed file formats (e.g., .wav; C. Balantic & T. Donovan, unpublished data).

Addressing these emergent acoustic monitoring challenges is crucial for building an expedient acoustic monitoring framework. As human land use and climate change continue to influence wildlife ranges and populations, there is a need to characterize status and trends of species that have been poorly understood and described (Thompson, 2004). Lacking a framework for optimizing acoustic sampling schedules amid constraints, landscape‐scale bioacoustic monitoring programs may fail to take full advantage of their monitoring efforts, resulting in compromised scientific inference and suboptimal conservation management decisions.

In this paper, we introduce a novel, temporally adaptive acoustic monitoring methodology for recording devices that can communicate remotely via Wi‐Fi or cellular network. Devices that can remotely transmit recordings are inherently equipped to receive external instructions about when to record on the following day. Our method optimizes these instructions across time and monitoring locations by tracking p*, also known as *p(capture)*, the probability of acoustically capturing (detecting) a target species at least once at any monitoring site at any time during the study (sensu Otis, Burnham, White, & Anderson, 1978; White, Anderson, Burnham, & Otis, 1982). Note that the concept of p*, the probability of capture, should not be confused with the concept of *p* in occupancy modeling, which indicates the probability of detection. By tracking p* for each species at each site on a daily basis, the timing of future acoustic surveys is allowed to vary across sites as a function of information from previous surveys. Once p* reaches a user‐defined threshold for target species at a given site, those species are released from future monitoring priority, allowing the recording schedule to focus more heavily on species that remain below target thresholds. Acoustic monitoring thus offers an opportunity to implement flexible, temporally adaptive sampling schedules that adjust automatically to optimize detection probabilities across a suite of focal species.

### 1.1 Objectives

The methodology described here was motivated by our real field experiences doing remote acoustic monitoring for a suite of focal species, using smartphones that transmitted audio files over the cellular network. The goal of this work was to design a temporally adaptive automated acoustic sampling algorithm and assess its potential for maximizing detection of multiple focal species. Specific objectives were to (a) develop a temporally adaptive automated acoustic sampling algorithm for acoustic wildlife monitoring subject to species prioritization and sampling constraints, (b) simulate hourly vocalization probabilities for nine species across 133 sites in a hypothetical monitoring area for the year 2016, and (c) implement a 2 × 6 × 2 factorial experiment to compare the probability of acoustic detection across sites in the 2016 vocalization simulation under differing monitoring protocols: schedule type (*n* = 2 levels; fixed schedule vs. optimized temporally adaptive schedule), sampling effort (*S* = 6 levels: 2, 5, 10, 20, 30, or 40 sampling minutes per day at each site), and monitoring duration (*D* = 2 levels: Full Year [*d* = 366 days for the 2016 leap year] vs. bird breeding season only [*d* = 31 days]).

## 2 MATERIALS AND METHODS

### 2.1 Objective 1: Develop an optimized adaptive sampling algorithm subject to species prioritization and sampling constraints

**Figure 1:**
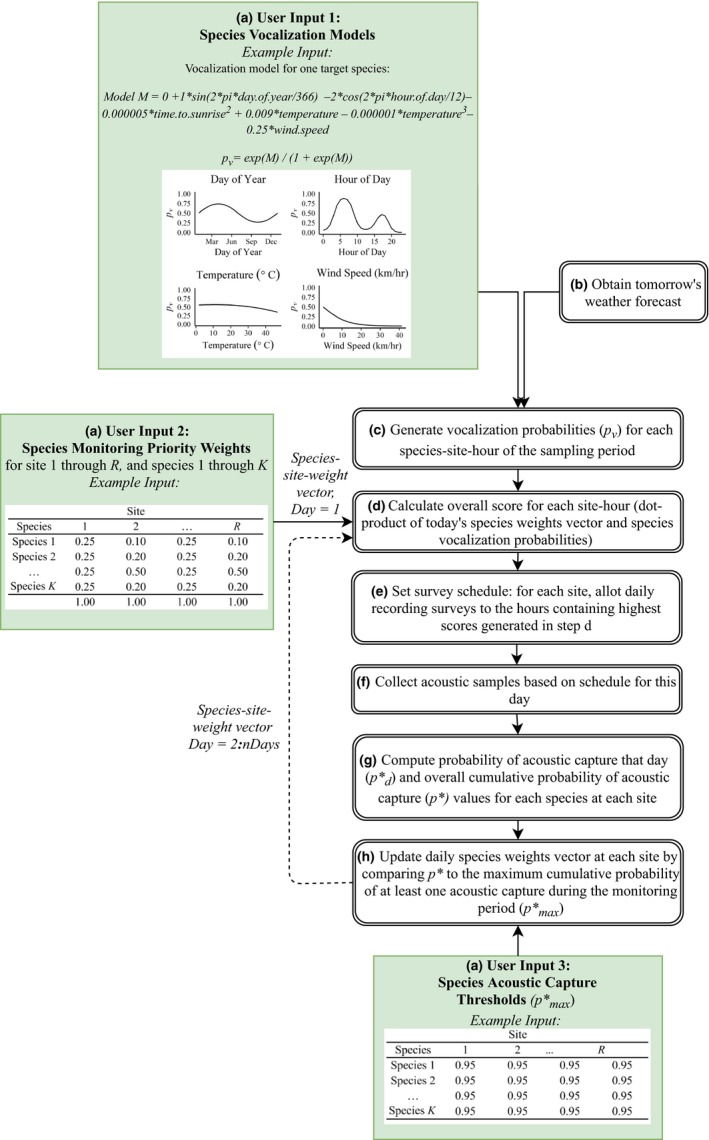
Objective 1 Workflow for an optimized temporally adaptive sampling algorithm subject to species prioritization and sampling constraints

We engineered a temporally adaptive sampling algorithm (Figure 1) designed to maximize detections across *K* target species and *R* study sites for *D* days, conditional on presence. The sampling schedule's unit of temporal adaptation was 1 day (i.e., the schedule updated every 24 hr and could not change mid‐day). In this approach, audio samples were collected on day *d*. Each day, based on these samples and forecasted temporal data, an optimized recording schedule was determined for the next day (*d* + 1).

Three fundamental user‐defined inputs provided the functionality for schedule optimization (Figure 1):

1. *Species vocalization models*: First, we created logistic regression vocalization models that reflected our knowledge about each of the *K* target species' vocalization patterns. We then used these models to predict the probability of vocalization (*p*_v_) for each species at each monitoring site during any hour of the day given existing weather and temporal conditions (User Input 1; Figure 1a).
2. *Species monitoring priority weights*: For each species in the focal group, we assigned an initial weight that reflected its user‐defined monitoring priority throughout the entire study period. Weights may be equal across focal species, or asymmetrical if a research program has varied species monitoring priorities and/or anticipates greater or lesser calling availability of certain species a priori (User Input 2; Figure 1a). The algorithm updated these weights on a daily basis as monitoring progressed.
3. *Species acoustic capture thresholds*: Third, for each species and site combination, we chose a monitoring threshold that informed the allocation of samples at each site. We designated this user‐defined monitoring threshold as *p* ^*^_max_, or the maximum cumulative probability of at least one acoustic capture during the monitoring period, if the species is present (Otis et al., 1978; White et al., 1982). For example, a *p* ^*^_max_ value of .95 for a given species at a given site indicated that monitoring should continue for this species at this site until the probability of detecting the species *at least one time* across the full monitoring period (*D*) met or exceeded .95 (i.e., monitor until p* ≥ *p* ^*^_max_; User Input 3, Figure 1a).

These three key inputs drove the optimized schedule (Figure 1), and utilized functions within the R package (R Development Core Team, 2019) *AMMonitor* on day *d* to produce the optimized recording schedule for each site on day *d* + 1. *AMMonitor* is a package intended to support adaptive management of biodiversity through remote monitoring methods and includes functions for automatic detection of target wildlife sounds with mitigation of false‐positive detections (Balantic & Donovan, 2019a), dynamic occupancy modeling from acoustic monitoring data (Balantic & Donovan, 2019b), and the temporally adaptive sampling algorithm described herein, which is implemented in the *AMMonitor* function *scheduleOptim()*.

For each day *d* of monitoring, we used *AMMonitor's* *temporalsGet()* function to obtain site‐specific, hourly weather forecast data for the next day (*d* + 1). We combined this temporal data with the species vocalization models to predict each species' hourly probability of vocalization (*p*_v_) at each site on day *d* + 1, hereafter “site‐hour” (Figure 1b,c). Next, the *AMMonitor* function *scheduleOptim()* calculated a single overall score for each site‐hour, computed as the dot‐product of the species weights vector and the species vocalization probabilities vector (Figure 1d). On day *d* = 1, the weights vector consisted of the species monitoring weights assigned at the start of the monitoring program (Figure 1a). In later iterations, it was a vector updated based on the probability of acoustic capture (p*) computed from previous sampling intervals (Figure 1h). The site‐hour scores were then ranked for each site, identifying the optimal hour(s) for sampling within each site for day *d* + 1. The *scheduleOptim()* function then scheduled *S* 1‐min samples, evenly spaced, into the highest scoring hour(s) for each site for day *d* + 1 (Figure 1e). The schedule was then sent to the recording unit, which collected audio samples as instructed the following day (Figure 1f). Based on the optimized recording schedules (which could vary from site to site) and the *p*_v_ associated with that hour for each species, we then computed *p* ^*^_d_ for each species at each site, where *p* ^*^_d_ was defined as the probability of detecting the species at least once that day given the sampling schedule (Figure 1g). We recomputed the cumulative probability of acoustic capture across *all* previous days (p*) for each species at each site at the end of each day (Figure 1g). The daily update of p* permitted priority weights of each species at each site to shrink or grow based on how likely it was that the species has already been adequately acoustically captured by previous sampling (Figure 1h). When p* equaled or exceeded our chosen *p* ^*^_max_ threshold at a given site, the species' updated weight at that site dropped to zero, allowing remaining sampling to emphasize species for which acoustic capture remained inadequate. The algorithm repeated daily until the sampling period *D* was complete or until all p* ≥ *p* ^*^_max_ for each species at each site.

### 2.2 Objective 2: Simulate hourly vocalization probabilities for nine species across 133 sites in a hypothetical monitoring area for the year 2016

#### 2.2.1 Study site

To test the utility of the algorithm, we simulated hourly vocalization probabilities for nine species across 133 sites for 366 days (2016 was a leap year), and then sampled from this acoustic environment in Objective 3. Our focal study area in this work was the Bureau of Land Management's (BLM) Riverside East Solar Energy Zone, a 599 km^2^ parcel allocated as a utility‐scale solar renewable energy hub in southeastern California, USA. The Riverside East Solar Energy Zone contains 133 sites actively monitored under an adaptive management protocol for vegetation indicators (Bureau of Land Management, 2016). We used these 133 sites as study locations for our simulation, to investigate the possibility of implementing temporally adaptive sampling in the field at a large scale.

#### 2.2.2 Study species

Based on literature and the monitoring interests of U.S. BLM, we selected nine study species for this simulation: Black‐tailed Gnatcatcher (*Polioptila melanura*), Common Poorwill (*Phalaenoptilus nuttallii*), Couch's Spadefoot (*Scaphiopus couchii*), Coyote (*Canis latrans*), Eurasian Collared‐Dove (*Streptopelia decaocto*), Gambel's Quail (*Callipepla gambelii*), Lesser Nighthawk (*Chordeiles acutipennis*), Phainopepla (*Phainopepla nitens*), and Verdin (*Auriparus flaviceps*). These species represented a mix of phylogenetic classes, diurnal and nocturnal vocalizers, early and late‐year vocalizers, common and uncommon vocalizers, residents and nonresidents, and species that are of conservation concern versus invasive species (Table 1).

**Table 1:** Summary of nine focal species used for simulation

Species	Species code	Phylogenetic class	Vocal availability throughout day	Vocal availability throughout year	Believed rarity of vocalizations, given presence	Resident or migratory	Native vs. Invasive
Black‐tailed Gnatcatcher	BTGN	Bird	Diurnal	Spring peak	Common	Resident	Native
Common Poorwill	COPO	Bird	Nocturnal	Spring peak	Common	Resident	Native
Couch's Spadefoot	TOAD	Amphibian	Nocturnal	Late summer/fall only	Rare	Resident	Native
Coyote	COYOTE	Mammal	Nocturnal	Peak at equinoxes	Uncommon	Resident	Native Invasive
Eurasian Collared‐Dove	ECDO	Bird	Diurnal	Spring peak	Common	Resident	Invasive
Gambel's Quail	GAQU	Bird	Diurnal	Spring peak	Common	Resident	Native
Lesser Nighthawk	LENI	Bird	Nocturnal	Spring peak	Uncommon	Migratory	Native
Phainopepla	PHAI	Bird	Diurnal	Spring peak	Common	Migratory	Native
Verdin	VERD	Bird	Diurnal	Spring peak	Common	Resident	Native

#### 2.2.3 Vocalization models

We used the *AMMonitor* function *simGlm()* to create literature‐based logistic regression models that predicted the probability of vocalizing at least once during a single hour of a given day for all nine target species (*p*_v_), conditional on presence. This function produced a statistical model of class “*glm*” (generalized linear model) in R. Model covariates for any given species included date, hour of day, lunar phase, and proximity to sunrise and/or sunset, as well as weather conditions such as temperature, wind, and precipitation. In the interest of simplicity, and because this method focused on the probability of acoustically capturing a species given presence, we did not include any spatial (habitat) covariates.

To accommodate the circular nature of temporal predictive variables like day of year, hour of day, and lunar phase, we modeled sine and cosine‐based coefficients. For example, we modeled hour of the day on a 24‐hour scale as sin(2*pi*hour.of.day/24) and cos(2*pi*hour.of.day/24). To provide finer control over the modeling outcome, we also modeled hour of the day on a 12‐hour scale as sin(2*pi*hour.of.day/12) and cos(2*pi*hour.of.day/12). To illustrate with a hypothetical example, the 0‐intercept model *M* describes the vocalization process of Eurasian Collared‐Dove (*Streptopelia decaocto*):

$$\begin{array}{c} M=0+1*\sin\left(2*pi*day.of.year/366\right)-2*\cos\left(2*pi*hour.of.day/12\right)-0.000005*\\ time.to.sunrise^{2}+0.009*temperature-0.000001*temperature^{3}-0.25*wind.speed \end{array}$$

The probability of vocalizing at least once during a given hour on a given day (*p*_v_) was subsequently obtained by applying the logit link function:

$$p_{v}=\exp\left(M\right)/\left(1+\exp\left(M\right)\right)$$

**Figure 2:**
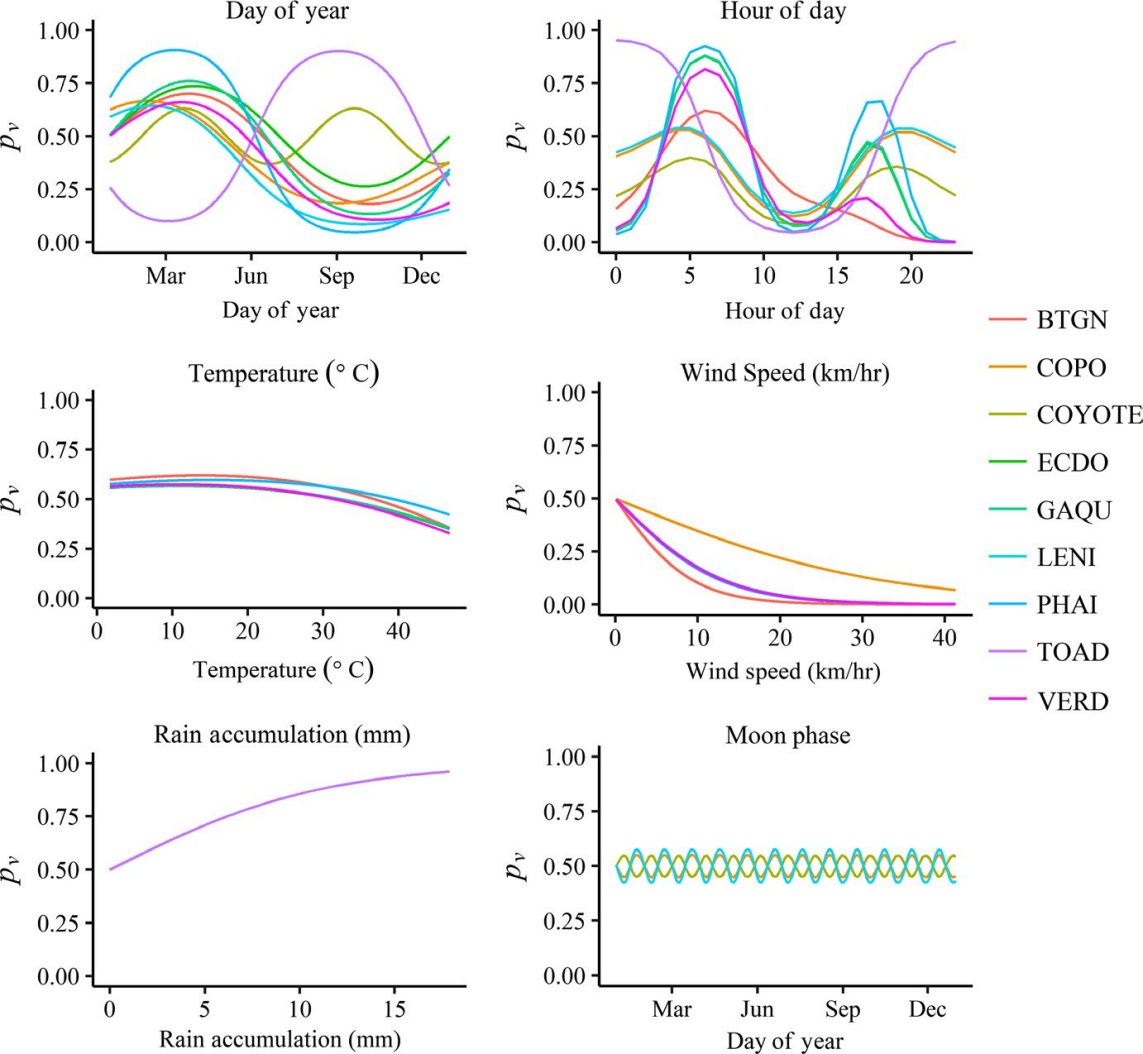
Visual demonstration of species logistic regression vocalization models. Species codes and regression models are given in Table 2. The probability of vocalization (*p*_v_), given presence, is graphed as a function of key weather and temporal covariates to display vocalization characteristics across species. Because covariates are graphed separately, intercepts of zero are used for visual demonstration purposes

We developed logistic regression models that reflected our literature‐based knowledge about vocalization activity for all nine focal species (Table 2). All models used some combination of distance to sunrise/sunset and/or circular temporal variables (day of year, time of day) modeled with sin and cosine. We visualized the impacts of these variables on each species' vocalization probability in Figure 2. Temperature and wind speed were included for all diurnal avian species (U. S. Geological Survey, 2001). The nocturnal avian species models included variables for wind speed and cosine of the lunar phase because vocal availability may be improved on moonlit nights (Woods, Csada, & Brigham, 2005). The coyote model also contained the cosine of the lunar phase because this species may be more vocally active at the new moon (Bender, Bayne, & Brigham, 1996). The Couch's Spadefoot model included rain accumulation within the past 24 hr (Mayhew, 1965). Based on the literature, we made Couch's Spadefoot, Coyote, and Lesser Nighthawk less vocally available and thus more difficult to detect (Table 2).

**Table 2:** Logistic regression models for nine focal species, each producing the hourly probability of vocalization

Species	Model
Black‐tailed Gnatcatcher (BTGN)	−0.3 − 0.002*day.of.year + 1*sin(*day.of.year*) − 0.5*cos(*hour*_12_) − 0.000007*time.to.sunrise ^2^ + 0.009*temperature – 0.000001*temperature ^3^ − 0.35*wind.speed
Common Poorwill (COPO)	−1.5 − 0.003*day.of.year − 0.5*cos(*day.of.year*) + 0.6*sin(*day.of.year*) + 1*cos(*hour*_24_) − 0.5*cos(*hour*_12_) − 0.0005*time.to.sunrise − 0.0005*time.to.sunset − 0.1*wind.speed − 0.2*cos(*lunar.phase*)
Couch's Spadefoot (TOAD)	−8 − 1*cos(*day.of.year*) − 2*sin(*day.of.year*) + 3*cos(*hour.of.day*_24_) + 5*rain.accumulation.in.past.24.hours
Coyote (COYOTE)	−3 − 0.5*cos(*day.of.year_equinox_*) + 0.2*sin(*day.of.year_equinox_*) + 1*cos(*hour*_24_) − 0.5*cos(*hour*_12_) − 0.001*time.to.sunrise − 0.001*time.to.sunset + 0.2*cos(*lunar.phase*)
Eurasian Collared‐Dove (ECDO)	−1.4 + 1*sin(*day.of.year*) − 2*cos(*hour*_12_) − 0.000005*time.to.sunrise ^2^ + 0.009*temperature – 0.000001*temperature ^3^ − 0.25*wind.speed
Gambel's Quail (GAQU)	−1.2 − 0.002*day.of.year + 1.3*sin(*day.of.year*) – 2*cos(*hour.of.day*_12_) – 0.000005*time.to.sunrise ^2^ + 0.009*temperature – 0.000001*temperature ^3^ – 0.25*wind.speed
Lesser Nighthawk (LENI)	−2 – 0.006*day.of.year + 0.4*cos(*day.of.year*) + 0.7*sin(*day.of.year*) + 1*cos(*hour*_24_) – 0.5*cos(*hour*_12_) – 0.0005*time.to.sunrise – 0.0005*time.to.sunset – 0.25*wind.speed – 0.3*cos(*lunar.phase*)
Phainopepla (PHAI)	−2.2 – 0.00001*day.of.year ^2^ + 0.7*cos(*day.of.year*) + 2.2*sin(*day.of.year*) – 2.5*cos(*hour*_12_) – 0.000004*time.to.sunrise ^2^ + 0.009*temperature – 0.000001*temperature ^3^ – 0.25*wind.speed
Verdin (VERD)	−0.5 – 0.004*day.of.year + 1*sin(*day.of.year*) – 1.5*cos(*hour*_12_) – 0.000007*time.to.sunrise ^2^ + 0.009*temperature – 0.000001*temperature ^3^ – 0.25*wind.speed

Note

Covariates: *day.of.year* = integer of the day of the year, from 1 to 366; *day.of.year_equinox_* = integer of the day of the equinox period, from 1 to 183; *hour_24_* = integer of the hour of the day, on a 24‐hour scale; *hour_12_* = integer of the hour of the day, on a 12‐hour scale; *time.to.sunrise* = real number denoting absolute value of the time from sunrise, in minutes; *time.to.sunset* = real number denoting absolute value of the time from sunset, in minutes; *temperature* = real number denoting the temperature in degrees Celsius; *wind.speed* = real number denoting the wind speed in kilometers per hour; *lunar.phase* = fractional part of the lunation number, ranging from 0 (new moon), 0.25 (first quarter moon), 0.5 (full moon), to 0.75 (last quarter moon), with ranges in between representing waxing or waning crescent or gibbous moons (Dark Sky, 2017); *rain.accumulation.in.past.24.hours* = amount of rain accumulated in the past 24 hr, in millimeters.

#### 2.2.4 Calculate *p*_v_ for each site‐hour for each species at each location

For each day of 2016, we acquired hourly weather data for all 133 study sites using the *AMMonitor* function *temporalsGet()*. This function utilized the Dark Sky API (Dark Sky, 2017) to provide hourly data for precipitation intensity, precipitation probability, temperature, dew point, pressure, wind speed, cloud cover, ultraviolet index, visibility, and ozone, as well as the daily sunrise time, sunset time, and lunar phase associated with each monitoring site. The function appended variables such as the absolute value of time to sunrise or sunset, predicted rain accumulation in the previous 24 hr, day of year, and hour of day, and the aforementioned circular sine and cosine‐based predictors. We supplied the finalized covariate dataset and the class *glm* vocalization models (*n* = 9) to *R*'s *predict()* function to generate the probability of vocalization (*p*_v_) for each species, at each location, during each hour for the year 2016 in its entirety. This resulted in a dataset consisting of 9 species * 133 sites * 24 hr * 366 days = 10,514,448 *p*_v_ records from which to sample in Objective 3.

### 2.3 Objective 3: Apply both the optimized schedule and fixed (stationary) sampling schedule to the simulated environment and compare performance of the optimized schedule and fixed schedule at different sampling efforts and study season lengths

We implemented a 2 × 6 × 2 factorial experiment that subsampled the Objective 2 vocalization simulation. The experiment consisted of two scheduling treatments (*Tr* = optimized or fixed) at six sampling effort levels (*S* = 2, 5, 10, 20, 30, or 40 min per day of sampling) and under two study durations (*D* = “Full Year (366 days)”: the full 2016 year using all nine species, and “March Only (31 days)”: a sole focus on the March 2016 breeding season, where most focal species were expected to be especially active and where Couch's Spadefoot was omitted because it was not expected to be active).

For the Full Year Optimization treatment, we applied our daily temporally adaptive sampling protocol beginning on January 1, 2016, and ending on December 31, 2016. For the March Only Optimization treatment, the temporally adaptive sampling protocol began on March 1, 2016, and concluded on March 31, 2016. In both cases, we set each initial *Species monitoring priority weight* to be equal at each site (1 divided by the total number of focal species; Table 3). Additionally, we selected *Species acoustic capture thresholds* (*p* ^*^_max_) of 0.95 for each species at each site.

**Table 3:** Monitoring priority weights for focal species at 133 sites, used for the Full Year (a) and March Only (b) study durations

Species	Site						
	1	2	3	…	131	132	133
(a)							
Black‐tailed Gnatcatcher (BTGN)	0.11	0.11	0.11	0.11	0.11	0.11	0.11
Common Poorwill (COPO)	0.11	0.11	0.11	0.11	0.11	0.11	0.11
Couch's Spadefoot (TOAD)	0.11	0.11	0.11	0.11	0.11	0.11	0.11
Coyote (COYOTE)	0.11	0.11	0.11	0.11	0.11	0.11	0.11
Eurasian Collared‐Dove (ECDO)	0.11	0.11	0.11	0.11	0.11	0.11	0.11
Gambel's Quail (GAQU)	0.11	0.11	0.11	0.11	0.11	0.11	0.11
Lesser Nighthawk (LENI)	0.11	0.11	0.11	0.11	0.11	0.11	0.11
Phainopepla (PHAI)	0.11	0.11	0.11	0.11	0.11	0.11	0.11
Verdin (VERD)	0.11	0.11	0.11	0.11	0.11	0.11	0.11
Sum	1.00	1.00	1.00	1.00	1.00	1.00	1.00
(b)							
Black‐tailed Gnatcatcher (BTGN)	0.13	0.13	0.13	0.13	0.13	0.13	0.13
Common Poorwill (COPO)	0.13	0.13	0.13	0.13	0.13	0.13	0.13
Coyote (COYOTE)	0.13	0.13	0.13	0.13	0.13	0.13	0.13
Eurasian Collared‐Dove (ECDO)	0.13	0.13	0.13	0.13	0.13	0.13	0.13
Gambel's Quail (GAQU)	0.13	0.13	0.13	0.13	0.13	0.13	0.13
Lesser Nighthawk (LENI)	0.13	0.13	0.13	0.13	0.13	0.13	0.13
Phainopepla (PHAI)	0.13	0.13	0.13	0.13	0.13	0.13	0.13
Verdin (VERD)	0.13	0.13	0.13	0.13	0.13	0.13	0.13
Sum	1.00	1.00	1.00	1.00	1.00	1.00	1.00

For the fixed treatment, we created stationary schedules for each sampling effort (*S*) (Table 4) in an effort to make them as competitive as possible with the optimized treatment at the same sampling effort. The *S* = 2‐min sampling effort consisted of a 1‐min sample in the morning (08:00:00) and a 1‐min sample at night (23:00:00). At higher efforts, samples were generally clustered around the average sunrise and sunset times throughout the year, with recordings scheduled on an hourly and subhourly basis as sampling effort increased. The same fixed schedules were applied for both the Full Year and March Only study durations.

**Table 4:** Fixed sampling schedules used on the 24‐hour clock at each sampling effort (*S* = 2, 5, 10, 20, 30, or 40 min), applied to both the March Only and Full Year study durations

Number of samples	Fixed schedule
2	08:00:00, 23:00:00
5	02:00:00, 05:00:00, 06:00:00, 08:00:00, 23:00:00
10	00:00:00, 01:00:00, 02:00:00, 06:00:00, 06:30:00, 07:00:00, 07:30:00, 08:00:00, 22:00:00, 23:00:00
20	00:00:00, 01:00:00, 02:00:00, 03:00:00, 04:00:00, 05:00:00, 05:30:00, 06:00:00, 06:30:00, 07:00:00, 07:30:00, 08:00:00, 18:00:00, 18:30:00, 19:00:00, 19:30:00, 22:00:00, 22:30:00, 23:00:00, 23:30:00
30	00:00:00, 01:00:00, 01:30:00, 02:00:00, 02:30:00, 03:00:00, 03:30:00, 04:00:00, 04:30:00, 05:00:00, 05:30:00, 06:00:00, 06:30:00, 07:00:00, 07:30:00, 08:00:00, 08:30:00, 09:00:00, 09:30:00, 10:00:00, 17:00:00, 17:30:00, 18:00:00, 18:30:00, 19:00:00, 19:30:00, 22:00:00, 22:30:00, 23:00:00, 23:30:00
40	00:00:00, 00:30:00, 01:00:00, 01:30:00, 02:00:00, 02:30:00, 03:00:00, 03:30:00, 04:00:00, 04:30:00, 05:00:00, 05:30:00, 05:45:00, 06:00:00, 06:15:00, 06:30:00, 06:45:00, 07:00:00, 07:15:00, 07:30:00, 07:45:00, 08:00:00, 08:15:00, 08:30:00, 08:45:00, 09:00:00, 09:30:00, 10:00:00, 17:00:00, 17:30:00, 18:00:00, 18:15:00, 18:30:00, 18:45:00, 19:00:00, 19:30:00, 22:00:00, 22:30:00, 23:00:00, 23:30:00

For the optimized treatment, the *scheduleOptim()* function allocated evenly spaced samples to the highest scoring hour(s) in 1‐min increments, with a buffer of at least 1 min between each sample. We settled upon this formulation as a consequence of real field testing within the Riverside East Solar Energy Zone, wherein we found that (a), schedules with a high number of sampling occasions mitigated the risk of individual events not being received and logged by remote audio recording devices and (b), smaller files produced by short recordings were more likely to be reliably dispatched over the cellular network. Thus, a maximum of 30 1‐min samples could be assigned to any single hour. For example, a sampling effort of *S* = 30 1‐min samples would allot all 30 evenly spaced samples, each 1 min in length, with a 1‐min buffer between each sample, into the highest scoring hour. For sampling efforts greater than 30 min (i.e., *S* = 40), additional minutes spilled over into the second highest scoring hour.

For each species, under each sampling effort (*S*) and study duration (*D*), we used two metrics to compare the performance of the optimized and fixed treatments. First, we rendered p* accumulation curves averaged across the 133 sites and computed the total area under these curves (AUC), with AUC values closest to 1 being best. We also calculated the average date *p* ^*^_max_ was achieved for each species across sites (if at all), on the assumption that earlier achievement dates were more desirable.

## 3 RESULTS

### 3.1 Vocalization simulation results (Objective 2)

Driven by weather and temporal covariates, the simulated environment produced hourly probabilities of vocalization for each of the nine species at each site for the entire year of 2016. Summary statistics of monthly temperature, 24‐hr rain accumulation, and wind speed demonstrated variation in weather covariates throughout the year, while sunrise and sunset times illustrated shifts in temporal covariates (Table 5), all of which showed differences in conditions between the March Only and Full Year study durations.

**Table 5:** Summary statistics for weather and temporal covariates across the simulation study area in 2016

Month	Temperature (°C)				24‐hour Rain accumulation (mm)				Wind speed (km/hr)			
	Avg.	Min.	Max.	*SD*	Avg.	Min.	Max.	*SD*	Avg.	Min.	Max.	*SD*
January	12.3	1.9	27.0	3.1	0.60	0.00	11.22	1.70	8.2	0.0	49.0	5.9
February	17.7	1.1	31.0	4.5	0.05	0.00	3.89	0.28	9.9	0.0	36.7	6.0
March	20.0	6.7	33.9	4.0	0.04	0.00	2.02	0.14	10.5	0.0	55.7	7.0
April	22.5	10.6	36.0	3.7	0.30	0.00	4.56	0.75	11.0	0.0	50.8	7.5
May	25.1	13.1	38.5	3.8	0.05	0.00	1.21	0.17	10.1	0.0	38.7	5.9
June	33.2	19.9	48.8	4.1	0.01	0.00	1.09	0.05	10.5	0.1	35.3	5.3
July	34.9	22.4	45.7	3.5	0.10	0.00	2.91	0.33	11.6	0.0	47.3	5.3
August	33.9	22.0	45.5	3.3	0.07	0.00	1.74	0.17	9.2	0.0	39.7	5.1
September	28.9	16.0	42.3	3.7	0.19	0.00	6.34	0.79	9.1	0.0	35.4	5.7
October	25.0	14.8	36.3	3.5	0.07	0.00	2.83	0.37	8.1	0.0	34.4	4.8
November	18.3	3.6	33.5	4.2	0.09	0.00	3.21	0.31	8.4	0.0	34.8	5.3
December	12.8	1.3	25.6	3.1	1.22	0.00	22.32	3.44	9.5	0.0	44.8	6.9

Month	Sunrise time				Sunset time			
	Avg.	Min.	Max.	*SD* (min)	Avg.	Min.	Max.	*SD* (min)
January	6:44:36	6:37:05	6:48:47	2.5	16:56:21	16:41:32	17:12:21	8.3
February	6:25:40	6:08:47	6:40:09	8.4	17:24:54	17:09:49	17:39:11	7.8
March^a^	6:26:22	5:53:20	6:55:34	20.2	18:26:43	17:36:28	19:02:57	35.2
April	6:09:42	5:51:13	6:29:42	10.5	19:12:48	19:00:05	19:25:48	6.6
May	5:40:29	5:30:27	5:53:45	6.0	19:36:04	19:22:52	19:48:35	6.6
June	5:31:31	5:28:40	5:35:58	1.3	19:52:58	19:45:17	19:57:58	2.9
July	5:43:09	5:32:53	5:54:46	5.6	19:50:56	19:40:32	19:57:53	4.2
August	6:04:36	5:51:58	6:16:55	6.4	19:26:09	19:06:34	19:43:29	10.0
September	6:25:35	6:14:02	6:37:09	5.9	18:47:07	18:25:05	19:08:51	12.1
October	6:47:37	6:34:16	7:01:57	7.2	18:06:39	17:48:02	18:27:16	10.7
November^b^	6:24:31	6:03:46	7:06:33	18.2	16:49:13	16:31:24	17:50:38	26.1
December	6:38:59	6:26:55	6:48:04	5.4	16:36:07	16:31:08	16:44:50	3.3

Note

Monthly summaries convey conditions in March as compared with the Full Year variation. Summaries include the average (Avg.), minimum (Min.), maximum (Max.), and standard deviation (*SD*) for each covariate.

a Begin daylight saving time.

b End daylight saving time.

**Figure 3:**
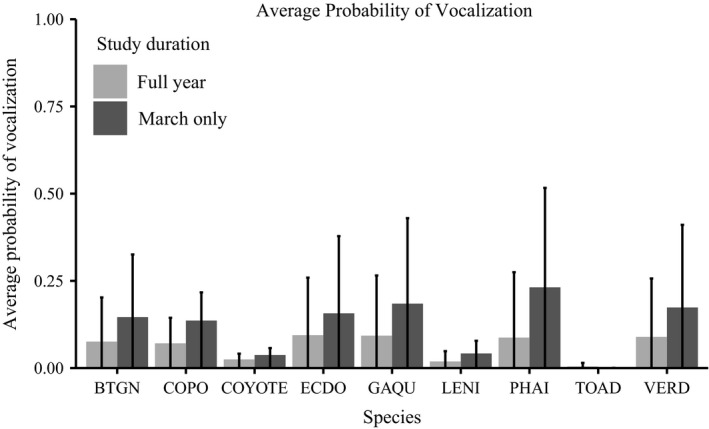
Vocalization Simulation Results. Average probability of vocalization in a given hour across all hours and sites for each focal species during both the March Only and Full Year study durations. Species codes are provided in Table 1. Standard deviation error bars reveal wide variation in vocalization probabilities contingent on weather and temporal conditions

The average probability of vocalization by species was summarized in Figure 3 for both study durations, showing that breeding birds had a higher average vocalization probability during the March study duration as compared to the entire year, and illustrating that three species—Couch's Spadefoot (TOAD), Coyote, and Lesser Nighthawk (LENI)—were far less vocally available in general than the other species. Large standard deviations in Figure 3 indicated the wide variation in overall vocalization probabilities across each hour of the year.

### 3.2 Factorial results (Objective 3)

Using the simulated environment for all species, the optimized treatment equaled or outperformed the fixed treatment on both metrics under all sampling efforts (*S*) and under both the Full Year and March Only durations (*D*), with only one exception (coyote *p* ^*^_max_ achievement at *S* = 20, *D* = Full Year).

In the optimized treatment, because we used equal initial monitoring priority weights for all species, gregarious species dominated the sampling allocation early on for both study durations. Species modeled to be more vocally available (Figure 3) initially had a greater effect on aggregate scores, causing sampling effort to be allotted in their favor early in the season. Once these species’ weights began shrinking as their p* values increased, optimized sampling focus shifted to less vocally available species.

**Figure 4:**
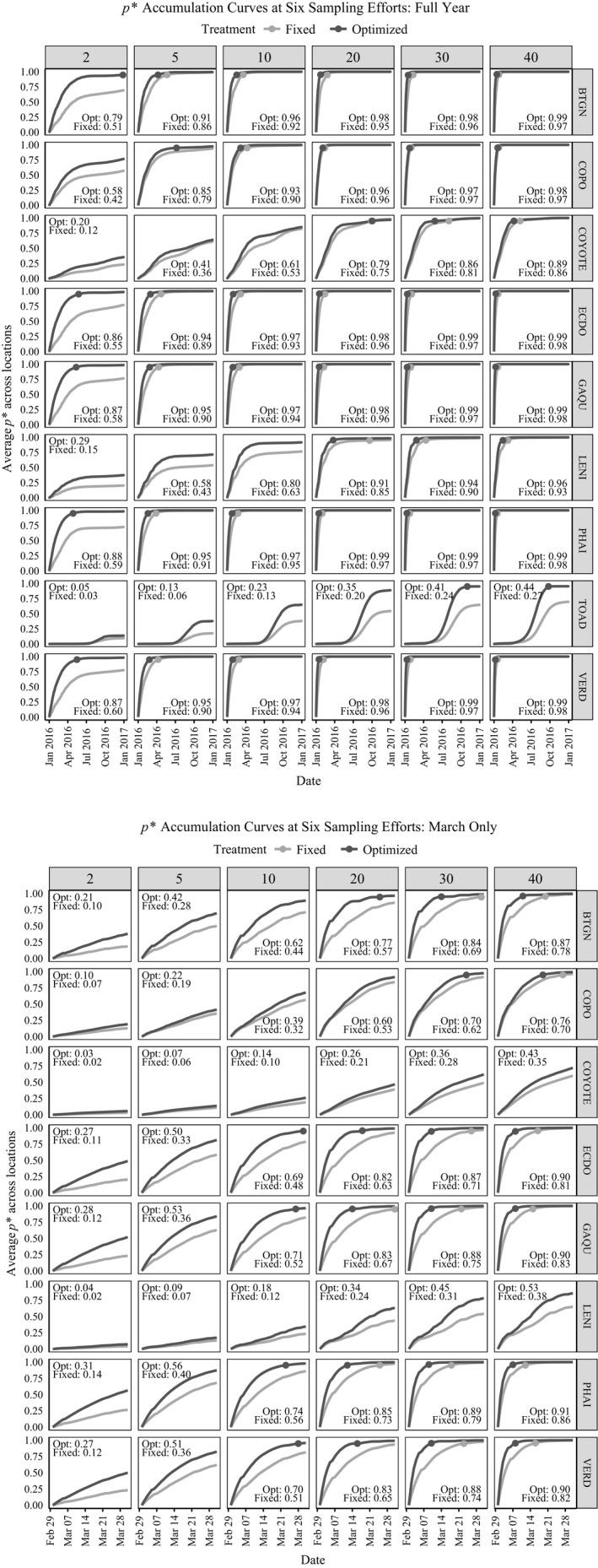
Factorial experiment results. p* and *p* ^*^_max_ charts are given for all focal species at six sampling efforts (*S = *2, 5, 10, 20, 30, or 40 1‐min samples) for the Full Year (a) and March Only (b) study durations. Species codes are provided in Table 1. Lines track cumulative p* values for both the fixed and optimized schedule treatments. Total area under the cumulative p* curve (AUC) values are given for both treatments within each box. Where applicable, the date of first *p* ^*^_max_ achievement is denoted by a single solid point on the line

Across species, AUC values produced by the p* accumulation curves for the optimized treatment equaled or exceeded those of the fixed treatment under all sampling efforts and for both study durations. At the extreme low end of sampling effort (*S* = 2 min per day), the optimized treatment yielded AUC values that were typically at least 25% greater than those of the fixed treatment during the Full Year study (Figure 4a), often ranging up to more than 50% greater for the March Only study (Figure 4b). Although the optimized AUC values were greater than the fixed AUC values in most cases, these differences became negligible for commonly available vocalizers during the Full Year study when sampling effort was high. For example, comparatively loquacious species, such as Black‐tailed Gnatcatcher, Common Poorwill, Gambel's Quail, Eurasian Collared‐Dove, Phainopepla, and Verdin attained relatively high AUC regardless of schedule type, provided that the study duration was sufficiently long and sampling effort was sufficiently high. Meanwhile, for the rarest vocalizers (e.g., Couch's Spadefoot), the optimized treatment substantially outperformed the fixed treatment AUC even when sampling was high over the longer study duration.

Schedules only achieved *p* ^*^_max_ values under certain conditions of sampling effort, study duration, and species vocal availability. For the Full Year study, where comparisons were possible, the optimized schedule reached *p* ^*^_max_ earlier in the year than the fixed schedule for nearly all scenarios (Figure 4a). The sole departure from this pattern was presented by the coyote, for which *p* ^*^_max_ was not obtained below a sampling effort of 20 min. At 20 min, both schedules attained *p* ^*^_max_ for coyote, although the fixed schedule reached this value 4 days earlier than the optimized schedule. In every other case, the opposite was true: across sites, for the Full Year study, the optimized schedule surpassed *p* ^*^_max_ anywhere from 5 to 179 days earlier than the fixed schedule depending on the species and sampling effort (average = 30 days earlier; Appendix 1). Even at 40 samples, where the fixed schedule began to become more competitive, the optimized schedule still reached *p* ^*^_max_ an average of 14 days earlier than the fixed schedule for all species except for Couch's Spadefoot, where no comparison was available because the optimized schedule achieved *p* ^*^_max_ and the fixed schedule did not (Figure 4a). In general, for both the fixed and optimized treatments, commonly available vocalizers (e.g., Eurasian Collared‐Dove, Gambel's Quail, Verdin) exceeded *p* ^*^_max_ earlier in the season than less available vocalizers (e.g., Coyote, Lesser Nighthawk), and the least available species (Couch's Spadefoot) only reached *p* ^*^_max_ with the optimized schedule. This outcome is consistent with simulated differences in average vocalization probability between species (Figure 3), given that we assigned equal initial monitoring priority weights to each species.

Under the abbreviated March sampling duration (where the Couch's Spadefoot was omitted due to seasonal inactivity), the optimized schedule again proved superior on the *p* ^*^_max_ metric (Figure 4b). Only six out of the eight species hit *p* ^*^_max_ at all during the shorter sampling season. Often *p* ^*^_max_ was achieved only at higher sampling efforts, even for commonly available vocalizers such as Eurasian Collared‐Dove, Gambel's Quail, and Verdin. In all cases, the fixed schedule lagged well behind the optimized schedule in attaining *p* ^*^_max_, if at all. For conditions under which a comparison was even possible, across all species and sampling efforts in the March Only study, the optimized schedule reached *p* ^*^_max_ an average of 11 days earlier than the fixed schedule (Appendix 1).

## 4 DISCUSSION

We demonstrated that a temporally adaptive optimized sampling schedule can substantially outperform a fixed schedule in a simulation setting for maximizing the probability of detecting a suite of focal species, given presence. The advantage of the optimized schedule was magnified especially for the shorter study season and particularly at lower sampling efforts. Simulation provided the opportunity to investigate the utility of temporally adaptive sampling on a limited budget and with a small pilot study sample size (*n* = 16) before attempting to implement this concept in the field at all actively monitored sites for ecological monitoring (*n* = 133). Depending on research questions and objectives, simulation can uncover whether temporally adaptive sampling is necessary for a given project. For certain programs and target species, temporally adaptive sampling will add no value, and false negatives can be adequately dealt with using occupancy modeling frameworks (e.g., if there are few target species or target species may be monitored sufficiently using a fixed schedule; if audio recording sampling capacity is high). For other circumstances, temporally adaptive sampling may enable monitoring that might otherwise have been impossible (e.g., if target species are only available under specific conditions; if there are constraints on the number and length of audio recordings that may be taken). Depending on the research question, a fixed schedule may be preferable if researchers prioritize comparability of sampling efforts over maximization of detection probabilities, because the temporally adaptive sampling routine may produce different daily recording schedules at different sites.

This work contributes novel methodology to the adaptive sampling paradigm for monitoring wildlife. The bulk of research on adaptive sampling of wildlife is focused on sampling in the spatial dimension (e.g., Thompson, White, & Gowan, 1998; Thompson, 2004; Turk & Borkowski, 2005), while temporal adaptive sampling has not been explicitly explored in great depth (though see Charney, Kubel, & Eiseman, 2015; Dyo et al., 2012). Recent work on the optimization of survey effort over space and time (Moore & McCarthy, 2016), and when species detectability varies (Moore, McCarthy, Parris, & Moore, 2014), explicitly incorporates the opportunity cost incurred by researchers when traveling to a field site for sampling; conceptually, the travel cost parameter may be framed as an analog to the costs of wireless data plans in remote acoustic recording units. Additionally, although the notion of time‐sensitive sampling is present in wildlife surveys—for example, by surveying during seasonally appropriate occasions for breeding amphibians, or on spring mornings during the dawn chorus for breeding birds—such sampling is not adaptive in nature unless information from prior surveys is incorporated into future sampling efforts (Charney et al., 2015; Thompson & Seber, 1994).

Accordingly, the adaptive nature of this methodology introduces new possibilities. A temporally adaptive sampling framework may be used to increase confidence in the local arrival and departure dates for migratory birds in a dynamic occupancy model framework (sensu MacKenzie, Nichols, Hines, Knutson, & Franklin, 2003; Miller et al., 2013; Balantic & Donovan, 2019b). Though occupancy models already account for detection errors in the form of false negatives, the adaptive optimization framework described here may reduce the false‐negative rate to provide more confidence in detection probability estimates. Additionally, a temporally adaptive approach may be useful for community‐level monitoring within multispecies occupancy models (Mackenzie, Bailey, & Nichols, 2004).

The optimization options developed here provide a framework for improved sampling granularity. First, in addition to local weather conditions, field‐based implementations of the temporally adaptive optimization scheme could incorporate real‐time bird migration predictions which combine citizen science observations via the eBird database (Sullivan et al., 2009), flight calls of nocturnal migrants, and radar to detect “clouds” of migrating birds (BirdCast: Cornell Lab of Ornithology, 2017). Given brief study durations, sampling constraints, and multiple focal species with varied vocal availabilities, automated optimization of acoustic sampling may thus allow research programs to collect higher quality data with limited resources.

Second, optimization methods might sample during the highest scoring time increments independent of site. In this work, we forced all sites to take *S* 1‐min samples daily, but future extensions could allocate all available sampling power within a given time period to the best “site‐hours” overall, perhaps across a 1‐, 3‐, or even 5‐day weather forecast. For example, if a study area is vast, and rain is forecasted for a subset of sites where Couch's Spadefoot is of high monitoring priority, available sampling power would be optimally distributed only to those site‐hours with high predicted rain accumulation. Rainless site‐hours, meanwhile, would be earmarked for no sampling during the forecast period of interest, minimizing wasteful sampling efforts if target species are only available under specific conditions.

Third, although this implementation optimized under an assumption of species presence, future extensions might set the adaptive schedule based on the joint probability of occupancy and vocalization. That is, our simulations set the optimized schedule based on the probability of calling, conditional on presence; we did not consider the factors that actually shape the presence or absence of species across the 133 sites. However, site occupancy can be factored into the algorithm by redefining *p*_v_ (currently, the conditional probability of vocalization given presence) as the joint probability of presence and vocalization. In this formulation, high presence probabilities produce a higher site‐hour score, increasing the chances of sampling a given site‐hour under the optimization scheme. In contrast, lower presence probabilities drive lower site‐hour scores, resulting in a smaller chance that a site‐hour will be selected for sampling.

Fourth, although this work focused on simulation results, in practice, researchers may incorporate additional considerations into a temporally adaptive sampling scheme implemented in the field. Firstly, vocalization models producing species vocalization probabilities (*p*_v_) may be generated such that they have confidence intervals that include upper and lower bounds. In practice, to accommodate model uncertainty, researchers may elect to use the upper bound, lower bound, or mean predicted *p*_v_ values in the optimization scheme, depending on model confidence. Secondly, although we used equal initial priority monitoring weights at all sites for all species, in practice, researchers maybe set higher weights for species or sites of greater monitoring priority. Thirdly, if the allocation of all sampling power into the highest scoring hour is undesirable due to low confidence in species vocalization models, researchers may explore alternative optimization schemes (Appendix 2).

Finally, although our *p* ^*^_max_ values were set to 0.95 for the simulation (i.e., sampling continued until there was a 95% chance the species was acoustically captured on our recording devices at least once), users may set this threshold to any value. For instance, we might relax the definition of *p* ^*^_max_ as a probability bounded between zero and one, and set a *p* ^*^_max_ value of 2.00 for a given species at a given site, which would indicate that monitoring should continue until we are quite confident that the species has been acoustically captured on at least two separate sampling occasions during the monitoring period. This arrangement could further safeguard against false negatives: first by providing an additional failsafe against recording at inopportune times, and second by adding preemptive cushion against false negatives that could occur as a consequence of using automated detection algorithms.

Although this work is simulation‐based, we field‐tested the mechanics of a temporally adaptive sampling optimization protocol on *n* = 16 audio recorders by connecting each Android audio recording unit with a site‐specific Google calendar account. We also developed a protocol linking the Android apps Easy Voice Recorder Pro (Digipom, 2016) and Tasker (Tasker, 2015) with the optimization protocol. This combination allowed us to populate each device's calendar with the optimized sampling schedule on a daily basis and collect acoustic recordings, providing a real field proof‐of‐concept for the simulation experiment detailed in this paper. This protocol can be implemented in the field using the fully operational *AMMonitor* functions *scheduleOptim()* and *scheduleFixed()*, which can be combined to create daily optimized and/or fixed schedules that are automatically pushed to a remote recording unit's Google account and then synced automatically for the next day of acoustic sampling.

## CONFLICT OF INTEREST

None declared.

## AUTHOR CONTRIBUTIONS

CB conceived the idea, analyzed and interpreted the data, wrote the code, and drafted the article. CB and TD collected data, designed methodology, revised the article critically for vital intellectual content, and gave final approval for publication.

## Data Availability

The *AMMonitor* package is available at https://code.usgs.gov/vtcfwru/ammonitor. R scripts for reproducing all manuscript results are available at https://github.com/cbalantic/temporally-adaptive-sampling.

## APPENDIX 1

Comprehensive results comparing the achievement of *p* ^*^_max_ (the maximum cumulative probability of at least one acoustic capture during the monitoring period) between the optimized sampling schedule and a fixed sampling schedule, for both the Full Year and March Only studies. The “Species” column refers to target species (see Table 1 for species codes). The “Optimized” column refers to the date upon which *p* ^*^_max_ was achieved using the temporally adaptive temporally adaptive sampling protocol outlined in the methods section of this paper. The “Fixed” column refers to the date upon which *p* ^*^_max_ was achieved using the fixed schedules denoted in Table 4. The “Difference (Days)” column indicates the difference in days of *p* ^*^_max_ achievement between the optimized and fixed schedules. Values of “–” indicate that *p* ^*^_max_ was not achieved, and no “Difference (Days”) comparison was possible. The “Effort” column denotes the sampling effort (*S* = 2, 5, 10, 20, 30, or 40 1‐min samples). The value in bold denotes the only case in which the fixed schedule achieved *p* ^*^_max_ prior to the optimized schedule.

**Table:**

Species	Optimized	Fixed	Difference (days)	Effort
Date *p* ^*^_max_ achieved (Full Year)				
BTGN	12/27/2016	–	–	2
ECDO	5/24/2016	–	–	2
GAQU	5/12/2016	–	–	2
PHAI	4/27/2016	–	–	2
VERD	5/16/2016	–	–	2
BTGN	4/7/2016	5/22/2016	−45.5	5
COPO	7/7/2016	–	–	5
ECDO	3/1/2016	4/22/2016	−51.5	5
GAQU	2/26/2016	4/10/2016	−43.8	5
PHAI	2/18/2016	3/28/2016	−38.7	5
VERD	2/25/2016	4/8/2016	−42.4	5
BTGN	2/17/2016	3/20/2016	−31.6	10
COPO	3/7/2016	4/8/2016	−32.5	10
ECDO	1/31/2016	3/7/2016	−35.7	10
GAQU	1/29/2016	3/1/2016	−32.3	10
PHAI	1/25/2016	2/23/2016	−28.7	10
VERD	1/28/2016	2/28/2016	−30.7	10
BTGN	1/23/2016	2/25/2016	−33.3	20
COPO	2/3/2016	2/13/2016	−10.8	20
COYOTE	10/2/2016	9/28/2016	**4.4**	20
ECDO	1/17/2016	2/13/2016	−27.2	20
GAQU	1/16/2016	2/8/2016	−23.4	20
LENI	3/25/2016	9/19/2016	−178.9	20
PHAI	1/14/2016	1/31/2016	−17.3	20
VERD	1/15/2016	2/6/2016	−22.7	20
BTGN	1/16/2016	2/9/2016	−23.9	30
COPO	1/23/2016	1/31/2016	−7.5	30
COYOTE	5/26/2016	8/3/2016	−68.8	30
ECDO	1/12/2016	2/2/2016	−21.4	30
GAQU	1/11/2016	1/29/2016	−18.1	30
LENI	2/26/2016	4/12/2016	−46.6	30
PHAI	1/10/2016	1/24/2016	−14.8	30
TOAD	11/1/2016	–	–	30
VERD	1/10/2016	1/27/2016	−16.3	30
BTGN	1/15/2016	1/25/2016	−10.1	40
COPO	1/19/2016	1/24/2016	−5.1	40
COYOTE	4/8/2016	5/8/2016	−30.0	40
ECDO	1/10/2016	1/21/2016	−11.3	40
GAQU	1/9/2016	1/19/2016	−10.5	40
LENI	2/14/2016	3/9/2016	−24.2	40
PHAI	1/8/2016	1/17/2016	−9.3	40
TOAD	9/24/2016	–	–	40
VERD	1/8/2016	1/18/2016	−9.4	40

**Table:**

Summary statistics for difference in days (Full Year)					
Min.	1st Qu.	Median	Mean	3rd Qu.	Max
−178.9	−34.5	−24.2	−30.0	−13.1	4.4

**Table:**

Species	Optimized	Fixed	Difference (days)	Effort
Date *p* ^*^_max_ achieved (March Only)				
No species achieved *p* ^*^_max_ below an effort of 10 samples				
BTGN	3/30/2016	–	–	10
ECDO	3/27/2016	–	–	10
GAQU	3/23/2016	–	–	10
PHAI	3/28/2016	–	–	10
VERD	3/25/2016	–	–	20
BTGN	3/18/2016	–	–	20
COPO	3/14/2016	3/31/2016	−16.8	20
ECDO	3/12/2016	3/25/2016	−13.2	20
GAQU	3/16/2016	–	–	20
PHAI	3/14/2016	3/30/2016	−16.0	30
VERD	3/24/2016	–	–	30
BTGN	3/10/2016	3/26/2016	−15.5	30
COPO	3/10/2016	3/22/2016	−12.3	30
ECDO	3/9/2016	3/18/2016	−9.2	30
GAQU	3/10/2016	3/23/2016	−13.9	30
PHAI	3/11/2016	3/20/2016	−9.0	40
VERD	3/19/2016	3/27/2016	−8.1	40
BTGN	3/8/2016	3/17/2016	−8.3	40
COPO	3/8/2016	3/15/2016	−7.1	40
COYOTE	3/7/2016	3/12/2016	−5.1	40
ECDO	3/8/2016	3/16/2016	−7.9	40

**Table:**

Summary statistics for difference in days (March Only)					
Min.	1st Qu.	Median	Mean	3rd Qu.	Max
−16.8	−13.9	−9.2	−11.0	−8.0	−5.1

## APPENDIX 2

### Comparison of simple optim versus max per hour versus fixed schedule

The main body of this paper used the simple daily site constraint option in *AMMonitor's* *scheduleOptim()* function, wherein an equal number *S* 1‐min samples per day were taken at each site and distributed into the highest scoring hour(s). Alternatively, samples may be distributed via the “max per hour” option. Using the “simple” daily site constraint option, if daily sampling effort is ≤30 min, all of those minutes are distributed at equal intervals into the highest scoring hour of the day. If this is undesirable, the researcher may invoke the “max per hour” option to specify a maximum number of samples that can be allocated into each hour. In an exploratory simulation using the March 2016 study duration (31 days), eight species, sampling efforts of *S* = 20, 30, and 40 1‐min samples per day, and a maximum number of samples per hr of 10, we found that the “simple” daily site constraint option outperformed the “max per hour” option slightly. The figure below demonstrates that the “simple” optimized schedule method, which preferentially allocates sampling power into the highest scoring hour, equals or outperforms the “max per hour” optimization method in the simulation, on both the *p** area under the curve (AUC) metric and on the achievement date metric for *p* ^*^_max_ (the maximum cumulative probability of at least one acoustic capture during the monitoring period). We provide the performance of the fixed schedule for comparison.
